# Could extended laminectomy effectively prevent spinal cord injury due to spinal shortening after 3-column osteotomy?

**DOI:** 10.1186/s12891-023-06751-w

**Published:** 2023-08-17

**Authors:** Yuyue Chen, Haozhi Yang, Ningling Xie, Shuang Zhang, Xiaobao Zou, Chenfu Deng, Binbin Wang, Hengrui Li, Xiangyang Ma

**Affiliations:** Department of Orthopedics, General Hospital of Southern Theatre Command, 111 Liuhua Road, Guangzhou, 510010 People’s Republic of China

**Keywords:** 3-Column osteotomy, Laminectomy extension, Prevention, Spinal cord Injury, Spinal shortening

## Abstract

**Objective:**

To explore whether the laminectomy extension can effectively prevent spinal cord injury (SCI) due to spinal shortening after 3-column osteotomy in goat models.

**Methods:**

A total of twenty healthy goats were included and done with 3-column osteotomy of T13 and L1 under the somatosensory evoked potential (SSEP) monitoring. The samples were divided into two groups. The first group underwent regular laminectomy while the second group underwent an extended laminectomy with an extra 10 mm-lamina cranial to L2. The SSEP measured after 3-column osteotomy was set as the baseline, and the SSEP decreased by 50% from the baseline amplitude and/or delayed by 10% relative to the baseline peak latency was set as positive results, which indicated spinal cord injury. The vertebral column was gradually shortened until the SSEP monitoring just did not show a positive result. The height of the initial osteotomy gap (the distance from the lower endplate of T12 to the upper endplate of L2), the shortened distance (△H), the number of spinal cord angulated and the changed angle of the spinal cord (△α) were measured and recorded in each group. Neurological function was evaluated by the Tarlov scores on day 2 postoperatively.

**Results:**

All the goats except one of the first group due to changes in the SSEP during the osteotomy were included and analyzed. In the first group, the height of the initial osteotomy segment and the safe shortening distances were 61.6 ± 2.6 mm and 35.2 ± 2.6 mm, respectively; the spinal cord of 5 goats was angulated (46.4 ± 6.6^°^), the other four goats were kinked and not angulated. In the second group, the height of the initial osteotomy segment and the safe shortening distances were 59.8 ± 1.5 mm and 43.3 ± 1.2 mm, respectively, and the spinal cord of ten goats were angulated (97.6 ± 7.2^°^). There was no significant difference in the height of the initial osteotomy segment between the two groups by using Independent-Samples T-Test, P = 0.095 (P > 0.05); there were significant difference in the safe shortening distance and the changed angle of the spinal cord between the two groups by using Independent-Samples T-Test (both $\Delta$H and $\Delta$α of P < 0.001), the difference between their mean were 8.1 mm and 51.2^°^. Significant difference was found in the number of spinal cord angulation between the two groups through Fisher’s exact test (5/9 vs. 10/10, P = 0.033).

**Conclusions:**

An additional resection of 10 mm-lamina cranial to L2 showed the satisfactory effect in alleviating SCI after 3-column osteotomy. Timely and appropriate extend laminectomy could be a promising therapeutic strategy for SCI attributable to facilitating spinal cord angulation rather than spinal cord kinking and increasing the safe shortening distance.

## Introduction

Spinal cord injury may occur due to excessive spinal shortening, spinal cord kinking and “pinch effect” from the posterior lamina after 3-column osteotomy process [1–4]. Lu et al. [1] reported that when the spinal cord is appropriately shortened, the tolerance of the spinal cord can increase and spinal cord injury resulting from angulation can be avoided. However, how to shorten the spine properly and how to make the spinal cord angulation properly have not been fully studied. Kadir et al. [5] reported that, during full-length shortening with total vertebrectomy and laminectomy of T12 in sheep cadaveric model, the mean kink of the spinal cord in the sagittal plane was 92.4^°^ for two-level hemilaminectomies of T11 and T13, 24.6^°^ for complete laminectomy of T11 with hemilaminectomy of T13, and 20.2^°^ for two-level complete laminectomies of T11 and T13, which indicated that it was possible to reduce excessive kinking of the spinal cord by performing a proper extended laminectomy in full-length shortening. Our previous studies [4] have found that during acute spinal shortening with bivertebral column resection and total laminectomy of T13 and L1 in live goat models, the safe shortening distances was 38.6 ± 1.2 mm and the corresponding changed angle of the spinal cord was 62.8 ± 6.9^°^ for laminectomy extension when performed on 10 mm caudal to T12, 41.5 ± 0.7 mm and 82.8 ± 7.5^°^ for laminectomy extension when performed on 5 mm caudal to T12 and 5 mm cranial to L2 simultaneously, 43.7 ± 0.8 mm and 98.5 ± 7.0^°^ for laminectomy extension when performed on 10 mm cranial to L2, which indicated that different laminectomy extension have different effects on the prevention of SCI and the enlarged resection of lower lamina could provide the biggest prevention efficacy for spinal cord injury. A laminectomy extension proximal and/or distal to osteotomy site is commonly performed to improve the safety of spinal cord. However, to perform a laminectomy extension after spinal shortening and spinal cord injured is not desirable for time demanding and increased surgical difficulty because of the obstruction of spinal cord in canal, which may cause adverse effect on spinal cord function recovery.

Enlightened by the above issues, we speculated that an extra resection of lamina prior to the spinal cord shortening and SCI might assist in spinal cord angulating and increasing the safe shortening distance, thus reducing damage to the spinal cord. To validate this speculation, we conducted the present study in which laminectomy extension was performed in living goat of SCI induced by spinal shortening after 3D-column osteotomy. The potential protective effect of such a treatment was evaluated by observing the spinal cord morphometry, electrophysiology and motor function.

## Materials and methods

### Surgical preparation

**Fig. 1:**
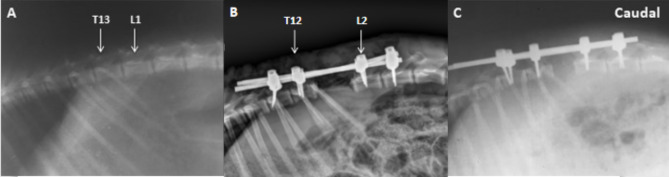
The pre- and postoperative X-ray films of T11-L3. (**A**) The preoperative X-ray film showed T13 marked at the 13th rib. (**B**) The postoperative X-ray film in the control group showed that the spinal column was shortened and the internal fixation was reliable. (**C**) The postoperative X-ray film in the second group showed that the spinal column was shortened and the shortened distance was longer than the first group

Twenty healthy adult goats (age, 20–26 months; body weight, 20–26 kg) were randomly divided into two groups. The first group with ten goats was the control group; the second group with ten goats (the laminectomy extension group): laminectomy extension was performed on 10 mm cranial to L2. All goats underwent fasting for 24 h prior to anesthesia. General anesthesia was performed with an injection of xylazine at dose of 0.15 mL/kg and 10mL of 3% pentobarbital sodium and was maintained by 3% pentobarbital sodium [6] 1.0 g cefazolin diluted into 100ml of 5% glucose solution was administrated intravenously to prevent infection. The arterial blood pressure was monitored through a cannula inserted into the carotid artery and was maintained stable by administering fluid. All goats were ventilated mechanically with air and body temperature was maintained between 36℃ and 37℃ by using a heating pad in the surgery. The desired segment of surgery (T11-L3) was previously marked using an X-ray apparatus (Fig. 1). All experiments were carried out with minimum suffering under the supervision of veterinarians at the animal laboratory center.

### Monitoring of SSEP

**Fig. 2:**
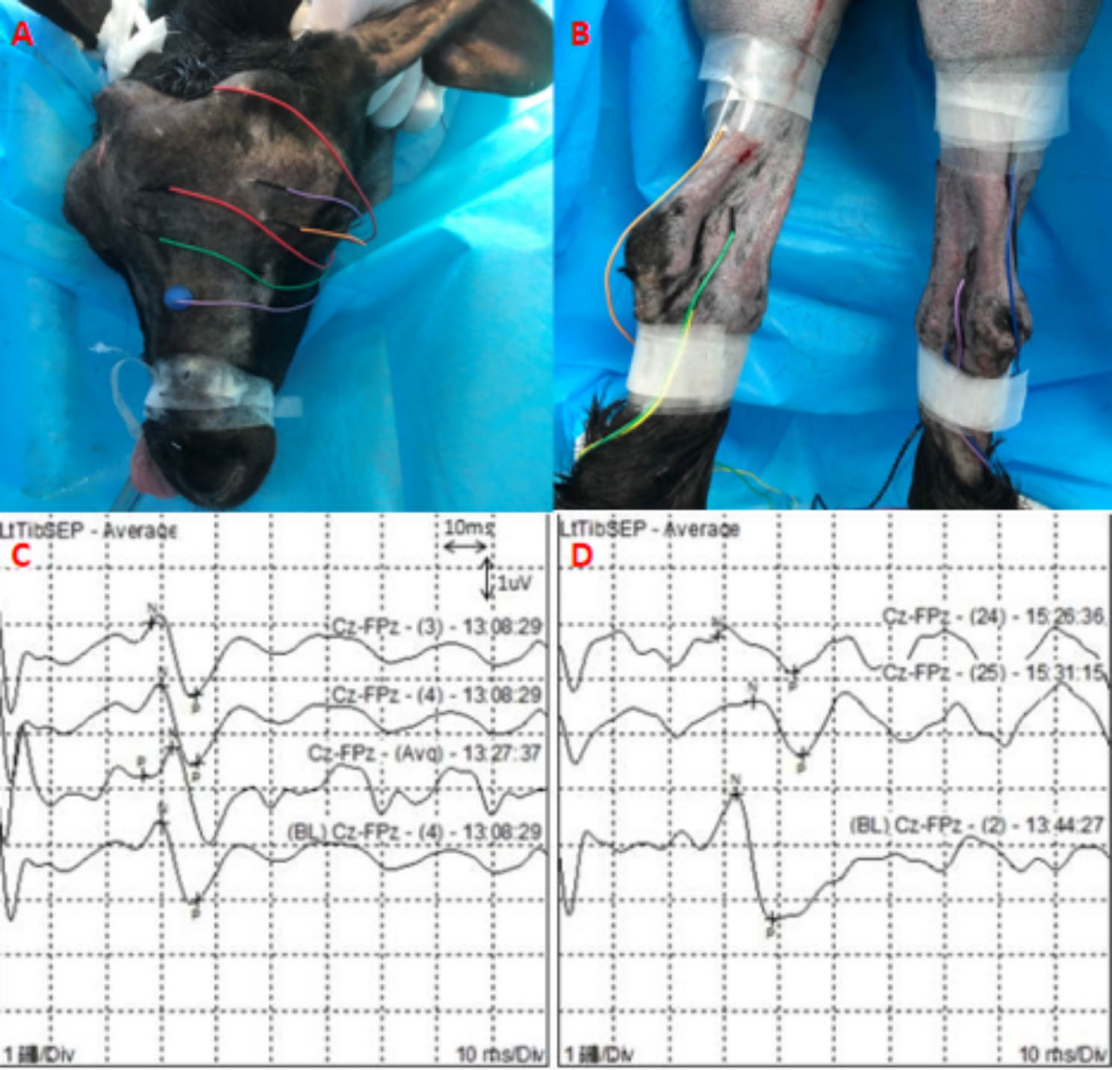
Somatosensory-evoked potential (SSEP). **(A, B)** The electrodes placement for SSEP. **(C)** The signals of SSEPs were under normal conditions. **(D)** The amplitude of SSEP decreased by 50%, and the peak latency of SSEP was delayed by 10%

The somatosensory-evoked potential, which is an electrical response signal in the scalp or spine evoked by stimulating the somatosensory afferent peripheral nerves, was implemented by a qualified electrophysiology technician using the Neuron-Spectrum System (Neurosoft; Cadwell, USA) to monitor the sensory pathway in real time during operation. The stimulating electrodes were placed on the posterior bilateral tibial nerves, and the recording electrodes were placed at ± 0.5 cm and 0.2 cm from the intersection of the ear lines and median sagittal line. The recording electrodes were placed on the left hindlimb sensory projection area of the cortex. A reference electrode was placed on the hard palate (Fig. 2A and B). The parameters of SSEP were as follows: stimulation intensity 20 mA, frequency 5 Hz, wave width 10 ms, superposition frequency 1000, filtering range 30-3000 Hz, sensitivity 0.5 Hz (Fig. 2C and D). The stimulus intensity remained constant during the whole monitoring process. A decrease in the amplitude of more than 50% from the baseline of the first positive wave and/or a delay of more than 10% relative to the baseline peak latency of the SSEP were considered a positive result [7, 8].

### Surgical procedure

**Fig. 3:**
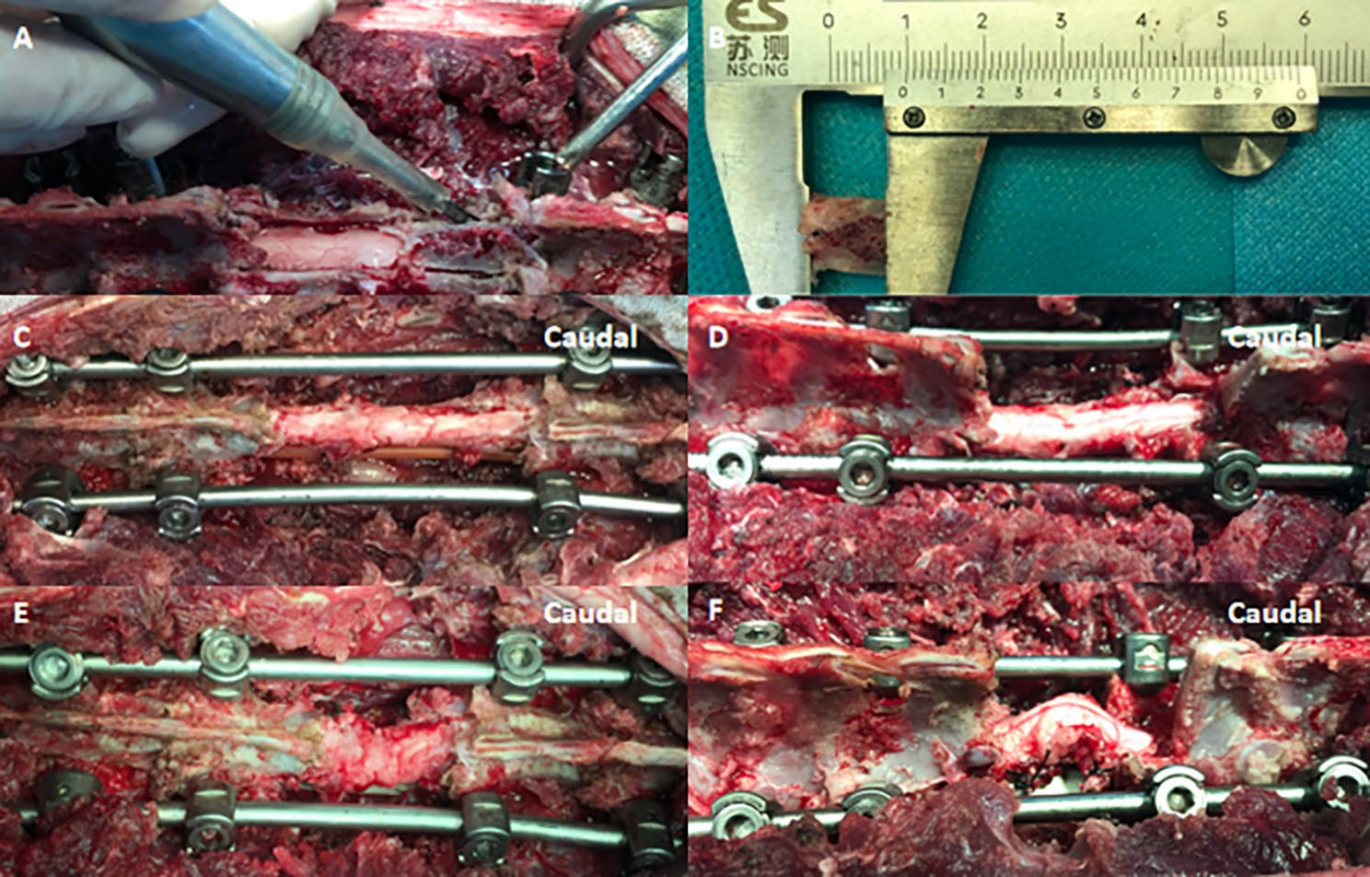
The experiments illustrating steps of each group. **(A)** A total laminectomy of T13 and L1 was performed by a piezosurgery. **(B)** In the second group, the pre-laminectomy extension of L2 was 10 mm. **(C)** In the first group, the 3-column osteotomy of T13 and L1 was completed and the spinal cord was in good condition. **(D)** In the second group, the pre-laminectomy extension was completed on 10 mm cranial to L2. **(E)** In the first group, the spinal cord tension increased and gradually kinked but not angulated in the sagittal plane. **(F)** In the second group, the spinal cord tension increased and gradually angulated in the sagittal plane

A midline longitudinal incision extending from T11 to L3 was performed, then the SSEP was recorded and set as the first baseline. The pedicle screws with 3.5 mm × 20 mm were inserted into the vertebrae of T11, T12, L2 and L3. The T11 and L3 levels were then stabilized unilaterally and temporarily with a suitable rod. A total laminectomy was performed from T13 to L1 (Fig. 3A). All parameters were measured by a vernier caliper (Fig. 3B). Then both nerve roots of T13 and L1 were ligated and removed. The T13 and L1 vertebral bodies and adjacent discs were resected completely using a piezosurgery (XD860A, SMTP, Suzhou, China). Subsequently, two suitable rods were assembled bilaterally and fixed firmly. Then, a laminectomy extension was performed on 10 mm cranial to L2 in the second group while no laminectomy extension was performed in the first group (Fig. 3C and D). The morphological changes of spinal cord during the spinal shortening process in each group were recorded (Fig. 3E F). Then, the SSEP was recorded and compared with the baseline amplitude. Any decrease in the SSEP amplitude or delay relative to the baseline peak latency during 3-column osteotomy were considered as an iatrogenic SCI, and these goats were excluded in the study. The SSEP after 3-column osteotomy was set as the final baseline.

**Fig. 4:**
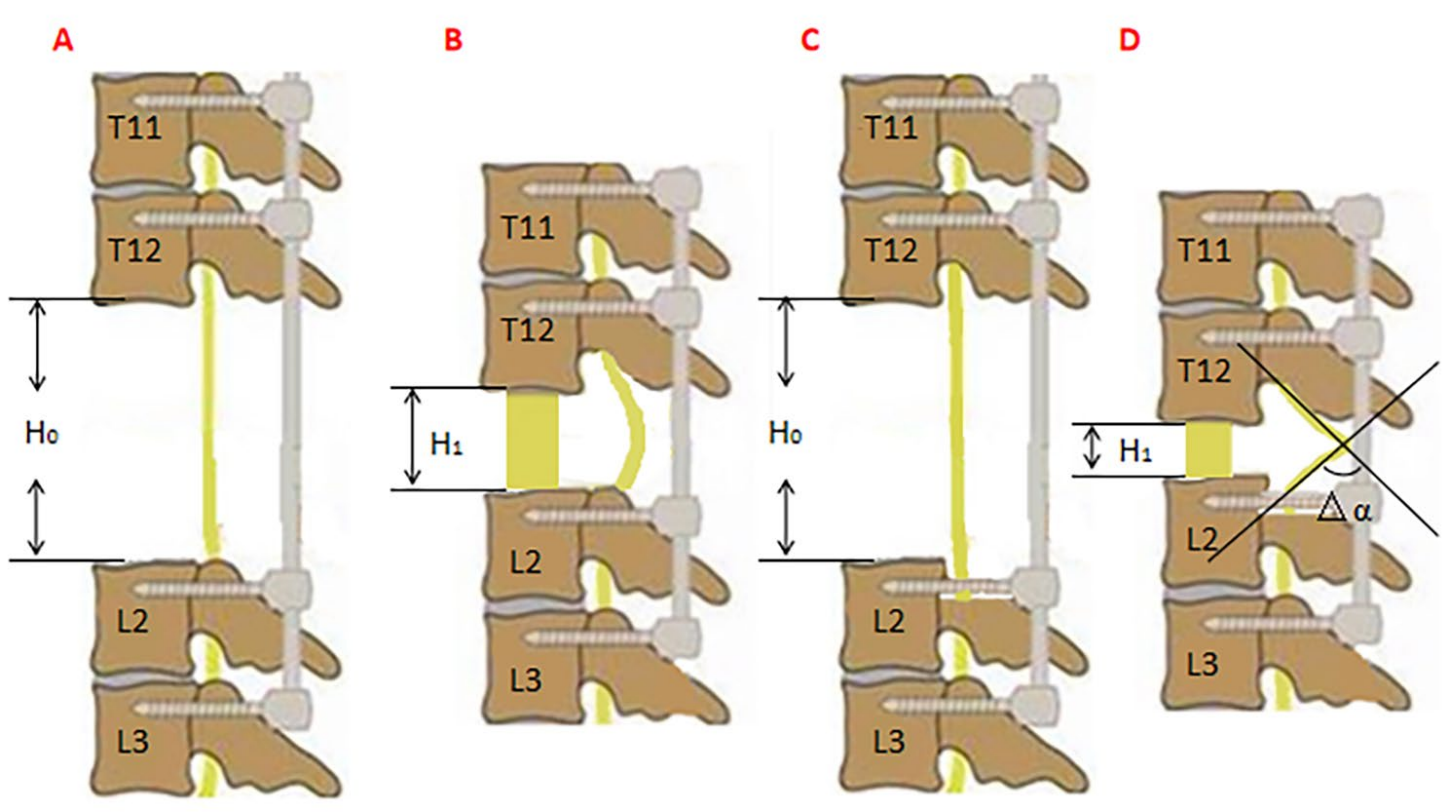
A series of images of the compression process in each group. **(A)** The first group: the pedicle screws were inserted into the vertebrae of T11, T12, L2 and L3, then 3-column osteotomy of T13 and L1 was completed. The initial osteotomy gap height (H_0_) was defined as the length from the lower endplate of T12 to the upper endplate of L2. **(B)** The first group: when the compression was ceased, the final osteotomy gap height (H_1_) was equal to the height of PEEK mesh cages and the spinal cord wasn’t angulated. **(C)** The second group: the pre-laminectomy extension was performed on 10 mm cranial to L2. **(D)** The second group: the spinal cord was angulated, H_1_ and the changed angle of the spinal cord (△α) was measured

The initial height of osteotomy gap (H_0,_ the distance from the lower endplate of T12 to the upper endplate of L2) was measured by a vernier caliper (Fig. 4A C). The vertebral column was shortened at the speed of 2.5 mm/10min by using a click-type stopper and the special PEEK mesh cages (The PEEK mesh cages are made by 2.5 mm height interval. The special cages are 15 mm, 17.5 mm, 20 mm, 22.5 mm, 25 mm, 27.5 mm, 30 mm, 32.5 mm, 35 mm, 37.5 and 40 mm, which can assist in compressing and preventing the spinal cord from moving forward). The vertebral column was gradually shortened until the SSEP monitoring just did not show a positive result, the final height of osteotomy gap (H_1,_ Fig. 4B and D) was equal to the height of the final cage. The shortening distance (△H = H_0_-H_1_), the number of spinal cord angulated and the changed angle of the spinal cord (△α, Fig. 4D) were measured and recorded in each group. This angle was the angulation between the proximal and the distal in the sagittal plane and was measured by goniometry. A wake-up test [9] was carried out to exclude false-negative results of SSEP, during which the motor function of these goats was evaluated. Subsequently, the pedicle screws were tightened at the rods after the goat was re-anesthetized. The postoperative neurological function was assessed by the Tarlov scores [10, 11] two days after surgery.

### Postoperative neurological function

On 2 days postoperatively, neurological function was assessed by the Tarlov scores: grade 0, complete paraplegia; grade 1, minor joint movements; grade 2, major joint movements; grade 3, could stand; grade 4, could walk; and grade 5, could climb a 20°-inclined plane.

### Data analysis

Statistical analysis was performed by using the SPSS 20.0. The differences of the height of the initial osteotomy segment (H_0_) and the shortening distances (△H) between the two groups were tested by Independent-Samples T Test, and P < 0.05 was considered statistically different. Fisher’s exact test was used to compare the differences of the number of spinal cord angulation between the two groups due to the total sample capacity was less than 40.

## Results

All the goats except one in the first group because of the alteration in SSEP signals during the 3-column osteotomy were included and analyzed. A meaningful change of SSEP was showed in the remained 19 goats, while the results of wake-up test of the remained goats were positive. On 2 days after operation, 3 goats were classified as Tarlov grade 4 and 6 as Tarlov grade 5 in the first group, 1 goat was classified as Tarlov grade 4 and 9 as Tarlov grade 5 in the second group. Postoperative radiograph showed the internal fixation was reliable (Fig. 1B C). The average operative time was 345 min (300 to 392 min), and the estimate blood loss was 96 ml (60–130 ml).

In the first group, the height of the initial osteotomy segment and the shortening distances were 61.6 ± 2.6 mm and 35.2 ± 2.6 mm, respectively; the spinal cord of 5 goats were angulated (46.4 ± 6.6^°^), the other four goats were not angulated. In the second group, the height of the initial osteotomy segment and the shortening distances were 59.8 ± 1.5 mm and 43.3 ± 1.2 mm, respectively, and the spinal cord of 10 goats were angulated (97.6 ± 7.2^°^). (Tables 1 and 2)

**Table 1:** The spinal parameters measured in the two group (H:mm, △α:^°^)

The first group						The second group				
No.	H_0_	△H	△α	Gd		No.	H_0_	△H	△α	Gd
1	61.4	36.4	54.0	5		1	60.2	42.7	93.0	5
2	65.4	35.4	/	4		2	58.6	43.6	96.0	5
3	58.1	33.1	38.0	5		3	57.8	42.8	89.0	5
4	60.0	35.0	45.0	5		4	59.7	44.7	107.0	4
5	63.9	38.9	52.0	5		5	61.9	44.4	102.0	5
6	59.2	31.7	/	4		6	58.9	43.9	104.0	5
7	59.0	31.5	/	5		7	59.1	44.1	105.0	5
8	62.8	37.8	43.0	5		8	58.3	40.8	95.0	5
9	64.3	36.8	/	4		9	61.7	41.7	85.0	5
10	/	/	/	/		10	61.4	43.9	100.0	5
Mean	61.6	35.2	46.4	/		Mean	59.8	43.3	97.6	/
SD	2.6	2.6	6.6	/		SD	1.5	1.2	7.2	/

NOTE. Gd: The postoperative neurological function was assessed by the Tarlov scores two days following surgery

/: means kinking

**Table 2:** Independent-Samples T-Test of the H_0_ and the △H

	t	df	Sig.(2-tailed)	Mean Difference	Std.Error Difference	95% Confidence Interval of the Difference	
						lower	Upper
H_0_	1.812	12.289	0.095	1.8067	0.9972	-0.3604	3.9737
△H	-8.436	11.190	0.000	-8.0822	0.9581	-10.1867	-5.9778
△α	-13.729	8.870	0.000	-51.20000	3.72946	-59.65551	-42.74449

Equal variances of H_0_ and △H were not assumed; Equal variances of △α was assumed

**Fig. 5:**
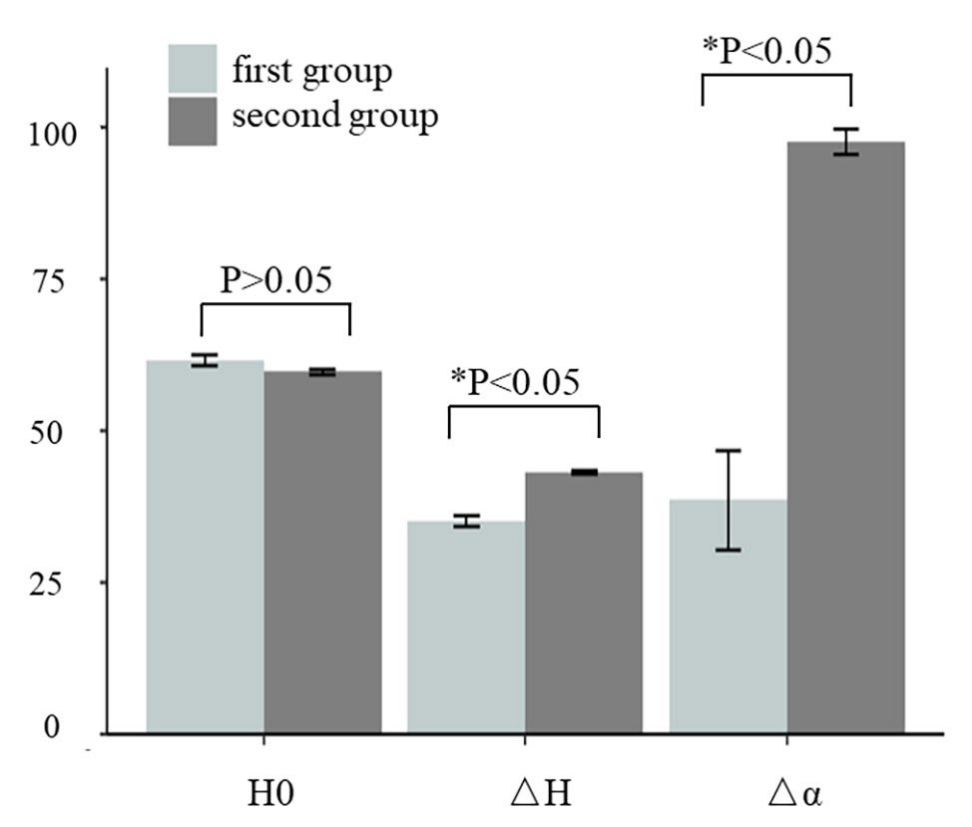
The comparison of H0, △H and △α between two groups were exhibited

There was no significant difference in the height of the initial osteotomy segment between the two groups by using Independent-Samples T-Test, P = 0.095 (P > 0.05); there were significant difference in the safe shortening distance (△H) and the changed angle of the spinal cord (△α) between the two groups by using Independent-Samples T-Test (both P < 0.001, Fig. 5), the difference between their mean were 8.1 mm and 51.2^°^ (Table 2). Significant difference was found in the number of spinal cord angulation between the two groups through Fisher’s exact test. (5/9 vs. 10/10, P = 0.033).

## Discussion

In this study, we explored the effect of extended laminectomy on SCI in goat models caused by spinal shortening by observing the morphometry, electrophysiologic assessment and motor function of the spinal cord. We found that the risk of kinking of the injured spinal cord was reduced and the angulation of the spinal cord occurred more frequently after the operation of extended laminectomy with an extra 10 mm-lamina cranial to L2 than regular laminectomy without additional lamina resection, resulted in a satisfactory recovery in SSEP and neurologic function. This result indicates that a prior appropriate extended laminectomy could be a promising therapeutic strategy for SCI caused by spinal shortening.

For the correction of some severe spinal deformities, 3-column osteotomy and spinal shortening are commonly required to achieve a satisfactory effect [12–15]. However, because of the compression of the lamina behind the spinal cord, there is not enough room for the spinal cord to move back sufficiently, so it could only move around the spinal canal to relieve the pressure, resulting in a wrinkled stack. When the decompression at the edge of the lamina, the caudal and rostral parts of the spinal cord moved backward, which alleviated the sharp kinking of the cord and resulted in an angulation. This is the anatomical basis for the validity of extended laminectomy in dealing with shortening-induced SCI. Although titanium mesh cage, interbody fusion cage or artificial vertebral body can be placed in the osteotomy gap after 3-column osteotomy to avoid excessively spinal shortening. It is still common for spinal cord compression to shorten the distance beyond the safe limit due to the different experience of the surgeon or to achieve the ideal orthopedic effect, which will cause excessive twisting of the spinal cord and consequent SCI. In order to reduce the spinal cord excessively kinking and prevent the aggravation of spinal cord injury, the routine therapy of clinical practice is loosening the rods and performing an additional laminectomy extension procedure. Our previous studies [16] have found that SCI was induced upon shortening by 106% of the vertebral height with single vertebral column resection (T10) in goat models where a total laminectomy was performed from T9-11. In addition, Kawahara et al. [17] reported that the so-called “dangerous range of shortening” was greater than two-thirds of the vertebral height with single vertebral column resection (T13) in dog models where a laminectomy was performed from the caudal region of T12 to the cranial regionof L1. Le Ji and colleges [18] concluded that an additional resection of two lamina at 6 h after shortening showed the best effect in alleviating SCI in dog models. These suggests that laminectomy extension can prevent SCI resulted from spinal shortening after 3-column osteotomy. However, the previous surgeons often performed the extended laminectomy after spinal cord shortening and SCI, which can adversely affect spinal cord function recovery due to the obstruction of the spinal canal, time requirements and increased surgical difficulty.

There are two factors meriting particular attention in an actual operation. The first is the additional length of lamina to remove. We can learn from Kadir’s experiment [5] that it was possible to reduce excessive kinking of the spinal cord by using the proper technique of laminectomy during full-length shortening in the sheep cadaveric model. But they didn’t elaborate on what the proper technique of laminectomy was and whether reducing excessive spinal cord kinking could prevent spinal cord injury, which is because spinal cord function cannot be monitored in the sheep cadaveric model. Subsequently, our previous studies [4] which was based on Kadir’s experiment [5] further found that different laminectomy extension after 3-column osteotomy has different effects on the prevention of SCI caused by acute spinal shortening in live goat models, and the enlarged resection of lower lamina is superior to equidistant enlarged resection of upper and lower laminas which is superior to enlarged resection of upper lamina in preventing SCI. However, our previous studies [4] did not establish a control group to elaborate on the difference between the laminectomy extension and non-laminectomy extension in the prevention of spinal cord injury caused by acute spinal shortening after 3-column osteotomy.

According to the result [4] that the enlarged resection of lower lamina could provide the biggest prevention efficacy for spinal cord injury, a distal laminectomy extension procedure was performed in the study group for significant difference. We can conclude from our present experiment that the laminectomy extension could facilitate spinal cord angulation which may relieve the pressure in the spinal cord, and increase the safe shortening distance and further effectively prevent spinal cord injury. This finding may be related to those factors that the laminectomy extension can effectively prevent the mechanical injury and hypoxic-ischemic injury of the spinal cord. First, the laminectomy extension can reduce the “pinch effect” of the posterior lamina and the rear vertebral body on the spinal cord, which gives the spinal cord more space in the back and promotes backward angulation of the spinal cord, thus reducing mechanical damage to the spinal cord. Then, the spinal cord angulated backward to avoid the spinal cord kinking and blocking the spinal cord blood flow, so as to reduce the hypoxic-ischemic injury of the spinal cord. Therefore, when spinal shortening is required after 3-column osteotomy, the appropriate laminectomy extension can be considered to prevent spinal cord injury.

The second factor is the time of laminectomy extension. Different from the study conducted by Le Ji [18], in our present study, we preformed the extended laminectomy prior to the start of spinal shortening for effective decompression management and provide the sufficient space for spinal cord to angulation rather than kinking due to the compression and the narrow space of the spinal canal. In the goat models, we found that performing the extended laminectomy in advance would effectively prevent the spinal cord kinking and increasing the safe range of spinal cord shortening. The results showed the increased safe shortening distances and the probability of spinal cord angulation after performing laminectomy extension surgery.

This experiment used healthy adult goats which have 13 thoracic vertebrae and 6 lumbar vertebrae. The structure and biomechanics of the goat spine has close similarities to the human spine [19]. The spinal cord of goat terminates near to the L6 level, while humans terminate at the L1-2 level, therefore the spinal cord at T13 and L1 levels in the goat models was not affected by the cauda equina nerve. All goats performed 3-column osteotomy of T13 and L1, pedicle screw-rod system and cages were placed to maintain the spinal stability, assist the spinal shortening. In our previous series of studies [4, 20], we found that the height of initial osteotomy gap is about 60 mm, the initial height of the cages is 40 mm, and the first shortened distance of the osteotomy gap is about 20 mm which is significantly smaller than the vertebral height. After first shortening, the shape of the spinal cord did not change significantly, and the SSEP was normal. Thus, the initial height of the cages was set at 40 mm. Moreover, in order to avoid delayed spinal cord injury during the surgery, the interval of compression was set at 10 min.

SSEP monitoring was used to evaluate sensory neural pathways continuously during the experiment, without obstructing the surgeon. However, a few false-negative and false-positive results have been reported, which are highly related to hypothermia, hypotension and inhalation anesthesia [21–23]. In this study, the goats’ body temperature and arterial pressure were closely monitored to eliminate the effects of low temperature and low blood pressure on SSEP. And we used intravenous anesthesia instead of inhalation anesthesia in the surgery. Moreover, no anesthesia drugs were used during acute spinal shortening in order to ensure the accuracy of SSEP monitoring [24]. Finally, to exclude false-negative and false-positive SSEP results, the wake-up test was used to evaluate the functional status of the spinal cord when the SSEP just did not appear a positive result.

The limitation of this study was that due to the limitations of experimental conditions and spinal cord tolerance, we did not make a comprehensive assessment of spinal cord during full-length spinal shortening, including the changes of spinal cord pressure and multimodal intraoperative monitoring, and the changes of spinal cord blood flow. Besides, in our study, the spinal column was shortened at a speed of 2.5 mm/10min, whether the spinal cord is safer and the result become more accurate if the spinal column was shortened at a slower speed like 2.0 mm/10min, or even 1.0 mm/10min was unclear. Additionally, for the small number of cases of this report, further study involving larger number of samples is required to explore the quantitative relationship between the range of laminectomy extension and spinal cord injury subsequent to acute spinal shorting after 3-column osteotomy.

## Conclusion

In goat models, the laminectomy extension after 3-column osteotomy can effectively prevent SCI in the process of immediately spinal shortening by facilitating spinal cord angulation rather than spinal cord kinking and increasing the safe shortening distance.

## Data Availability

The data used and analyzed during the current study are available in anonymized form from the corresponding author on reasonable request.
